# Public health failure in the prevention of neural tube defects: time to abandon the tolerable upper intake level of folate

**DOI:** 10.1186/s40985-018-0079-6

**Published:** 2018-01-31

**Authors:** Nicholas J. Wald, Joan K. Morris, Colin Blakemore

**Affiliations:** 1 Wolfson Institute of Preventive Medicine, Barts and the London School of Medicine and Dentistry, Queen Mary University of London, Charterhouse Square, London, EC1M 6BQ UK; 2 Centre for the Study of the Senses, School of Advanced Study, University of London, London, UK

**Keywords:** Folic acid, Folate, Neural tube defects, Spina bifida, Anencephaly, Tolerable upper intake level

## Abstract

The neural tube defects anencephaly and spina bifida are two of the most common serious congenital malformations. Most cases can be prevented by consuming sufficient folic acid immediately before pregnancy and in early pregnancy. Fortification of flour with folic acid to prevent these defects has been implemented in 81 countries without public objection or indication of harm. An obstacle to the wider adoption of fortification arises from the creation of a “tolerable upper intake level” for folate (which includes natural food folate as well as synthetic folic acid), and which has been set at 1 mg/day, thereby proscribing higher folate intakes. Increasing the intake of folic acid in a population will necessarily increase the number of people with a folate intake greater than 1 mg per day, and this concern is obstructing folic acid fortification. This paper shows that the scientific basis for setting any upper limit, let alone one at 1 mg/day, is flawed. An upper intake level is therefore unnecessary and should be removed, thus allaying unjustified concerns about folic acid fortification. As a result, the full global opportunity to prevent two serious fatal or disabling disorders can and should be realized.

## Background

Anencephaly and spina bifida, collectively known as neural tube defects (NTDs), are two of the most common serious birth defects throughout the world. They occur when the neural tube fails to close (closure is typically complete by 4 weeks after conception). About 1–2 in every 1000 pregnancies are affected and are either terminated following prenatal screening and diagnosis, or result in the birth of individuals with fatal or severely disabling malformations. It is tragic that, while NTDs can be prevented by increasing the consumption of folic acid in the population, this is not being achieved in practice in many parts of the world.

Folic acid (pterylglutamic acid (PGA), a vitamin in the B group) is the “core” part of a range of molecules collectively described as folate. Folic acid itself, which is not found naturally in food, becomes biologically active after metabolic reduction. It is a synthetic molecule that is used in pills (vitamin supplements) and can be added to staple foods such as flour (food fortification). Folic acid is ideal for use in this way because it is more stable, and about twice as bioavailable, as food folate; its stability means it is not degraded in cooking.

## The preventive effect

**Fig. 1:**
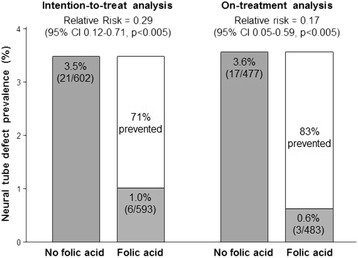
Results of MRC randomised trial of folic acid in the prevention of neural tube defects [1]

In 1991, the results of a randomised double-blind trial showed that NTDs are a vitamin deficiency disorder and that consuming 4 mg of folic acid daily immediately before and during the first trimester of pregnancy prevented about 80% of cases [1, 2]. The left part of Fig. 1 shows the trial results using an ‘intention-to-treat’ analysis (NTD rates in pregnant women allocated to take folic acid capsules compared with the rates in women allocated to take capsules that did not contain folic acid, which ensures the avoidance of selection bias). The right part of Fig. 1 shows the results using an ‘on treatment’ analysis (NTD rate in women who actually took folic acid capsules before pregnancy compared with the rate in women who took capsules that did not contain folic acid). This demonstrates the direct preventive effect, an interpretation that can be accepted as unbiased given the similar results from the intention-to-treat analysis.

A conclusion from the results of the randomised MRC vitamin study [1] is that neural tube defects are a vitamin deficiency disorder and accordingly require appropriate correction, both at the individual level with the use of folic acid supplements started before pregnancy and at the population level through folic acid fortification of a staple food such as flour. Folate deficiency is one example of a wider problem of specific global micronutrient deficiencies [3].

## Pre-pregnancy folic acid supplement use

**Fig. 2:**
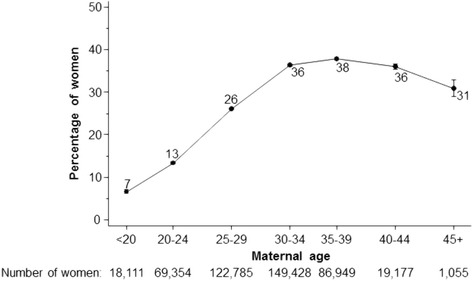
Percentage of pregnant women who took folic acid supplements before pregnancy according to maternal age (1999–2012). Percentages adjusted for year screened, maternal weight, ethnicity, previous NTD pregnancy, previous Down’s syndrome pregnancy, IVF, diabetes, smoking, Down’s syndrome screening test, and region of England. [4]

**Fig. 3:**
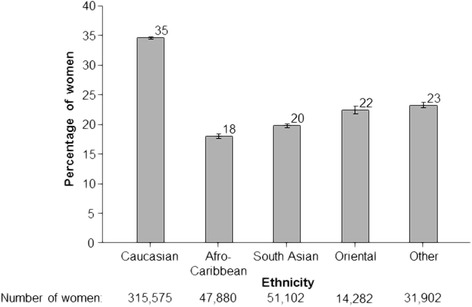
Percentage of pregnant women who took folic acid supplements before pregnancy according to ethnicity among women who provided this information (1999–2012). Percentages adjusted for year screened, maternal age, maternal weight, previous NTD pregnancy, previous Down’s syndrome pregnancy, IVF, diabetes, smoking, Down’s syndrome screening test, and region of England [4]

Despite vigorous campaigning by public health authorities, many women do not take folic acid supplements before pregnancy. A study based on nearly half a million women in England [4] showed that only 31% of women took folic acid supplements immediately prior to pregnancy; 62% started taking folic acid after pregnancy had been confirmed (but too late to prevent NTDs) and 8% did not take folic acid at all in early pregnancy. The proportion taking supplements before pregnancy was particularly low in young women (Fig. 2) and in non-white ethnic groups (Fig. 3). Even in the 680 women who had had a previous NTD pregnancy, and therefore should have been under medical care with the clear recommendation that folic acid supplements should be taken prior to pregnancy, fewer than half (47%) did so [4]. Thus, while all women should be encouraged to take folic acid supplements before pregnancy, as a practical public health measure, this strategy has substantial limitations and is putting young women and those in ethnic minorities at a particular disadvantage.

## Mandatory folic acid fortification

Eighty-one countries have introduced mandatory folic acid fortification of flour [5] (see Table 1), and all studies of the consequences have shown a clear reduction in the incidence of NTDs [6–8]. Expert committees have recommended fortification, but no European Union country has yet implemented the measure and European NTD rates have not declined between 1991 and 2011 [9]. The reluctance to introduce a public health intervention that would prevent death and disability is hard to understand. It seems that some expert committees undervalue the benefit. For example, the European Commission Scientific Committee on Food [10] referred to a low folate level or intake as a “risk factor for NTD risk” instead of recognizing it as an important cause of NTDs. Also, the prevention of NTDs does not feature highly on political agendas. In some countries, there is a view that public health is more about encouraging personal choice and changing “lifestyles” than about exercising collective choice and judgment.

**Table 1:** Countries with mandatory folic acid fortification (as of October 2017) [5]

Antigua and Barbuda	Ghana	Nigeria
Argentina	Grenada	Oman
Australia	Guatemala	Palestinian Authority Territory
Bahamas	Guinea	Panama
Bahrain	Guyana	Papua New Guinea
Barbados	Haiti	Paraguay
Belize	Honduras	Peru
Benin	Indonesia	Qatar^a^
Bolivia	Iran	Saint Kitts and Nevis
Brazil	Iraq	Saint Lucia
Burkina Faso	Jamaica	Saint Vincent and the Grenadines
Burundi	Jordan	Saudi Arabia
Cameroon	Kazakhstan	Senegal
Canada	Kenya	Sierra Leone
Cape Verde	Kiribati	Solomon Islands
Chile	Kosovo	South Africa
Colombia	Kuwait	Suriname
Costa Rica	Kyrgyzstan	Tanzania
Cote d’Ivoire	Liberia	Togo
Cuba	Mali	Trinidad and Tobago
Djibuti	Mauritania	Turkmenistan
Dominica	Mexico	Uganda
Dominican Republic	Moldova	United Arab Emirates^a^
DR Congo^a^	Morocco	United States of America
Ecuador	Mozambique	Uruguay
Egypt	Nepal	Uzbekistan
El Salvador	Nicaragua	Yemen
Fiji	Niger	Zimbabwe

^a^Voluntary fortification with most flour fortified

This view has driven a preference for the use of pre-pregnancy folic acid supplements instead of folic acid fortification, when they are, in fact, complementary interventions: neither is a complete substitute for the other. Fortification alone does not achieve full protection, but it provides a population safety net that contributes to the overall preventive effect, particularly important for the large number of women who have not taken supplements before becoming pregnant [11]. There is perhaps a reluctance to adopt a public health intervention when those individuals most likely to benefit could take appropriate action to protect themselves, but this argument denies much of what makes a society civilized and caring. The introduction of a legal requirement to use car seat-belts and the ban on smoking in public places show that responsible governments can and do act to reduce the risk of harm to others, even if personal freedoms are reduced. This principle is already established in the area of food supplementation for health benefit. Indeed, in the United Kingdom, flour is already mandatorily fortified with other B vitamins (thiamin and niacin), as well as with iron and calcium. Extending fortification to include folic acid would undoubtedly save many babies from the tragedy of being born with an NTD. Moreover, it would also benefit a sizeable fraction of the rest of the population, namely, those with folate deficiency, which itself is a cause of anaemia. In the USA, folate deficiency anaemia has been nearly completely eliminated following mandatory folic acid fortification of cereals [12]. The US National Health and Nutrition examination survey found that before the introduction of mandatory folic acid fortification, among people aged 65 and over with anaemia, 8.4% were due to folic acid deficiency (6.4% alone plus 2.0% due to folate and B12 deficiency) [13]; after fortification, this had fallen to 0.4% [12]. In the United Kingdom, some 6% of over 60s are folate-deficient based on a serum folate below 5 nmol/L (2.2 ng/ml) [14]. The prevalence of folate deficiency in the general population has decreased in countries that have introduced fortification. Thus, the value of fortification is wider than the prevention of NTDs.

With such evidence, the imperative for government to implement fortification is overwhelming. Only unequivocal evidence of harm could weigh against this decision. Unfortunately, a flawed assessment of potential harm is impeding the introduction of this life-saving policy.

### Is folic acid toxic?

The Dietary Reference Intakes (DRIs) developed by the Food and Nutrition Board at the Institute of Medicine (IOM) of the National Academies (formerly National Academy of Sciences) in the USA [15] include a recommended intake for folate, which has been widely accepted, including by the UK Scientific Advisory Committee on Nutrition (SACN 2017) [16]. The IOM found no evidence of harm from folic acid or food folate in respect of toxicology, reproductive, and developmental health or cancer. However, the IOM considered the possible exacerbation of neuropathy in individuals with vitamin B12 deficiency, treated with folic acid instead of B12, as a harm. They attempted to set a tolerable upper intake level (UL) for folate, defined as “The largest daily intake of a nutrient that is considered unlikely to cause harmful side effects for most people in a particular life stage and gender group” [15]. The UL is arbitrarily taken as one-fifth of the “lowest-observed-adverse-effect level” (LOAEL).

For folate, the IOM attempted to determine an LOAEL from a review of 23 studies of patients with a B12 macrocytic anaemia (pernicious anaemia), mainly conducted in the 1950s, 11 of which were single-patient case reports [15]. At that time, the distinction between folate deficiency and B12 deficiency was not recognised and assays for the two vitamins had not been developed. A deficiency in either vitamin causes the same type of anaemia— a macrocytic anaemia with a megaloblastic bone marrow. A patient with B12 deficiency may superficially appear to be treated successfully with folic acid because the macrocytic anaemia can resolve, but not the neurological disease. Only B12 administration will stop the subacute combined degeneration of the spinal cord and peripheral neuropathy. Intake of folic acid was, then, said to “mask” the diagnosis of B12 deficiency because folic acid resolves the anaemia. However, masking is now a misleading term to describe the clinical situation, because it reflects a historical period when folate deficiency could not be distinguished from B12 deficiency. Folic acid was used as treatment, and the macrocytic anaemia due to B12 deficiency remitted; the subsequent occurrence of a neurological deficit was wrongly interpreted as an adverse effect of folic acid instead of an inability to make the correct diagnosis and provide the necessary B12 treatment. Dickinson [17], who considered these reports, concluded that making an error of diagnosis should not be confused with possible folic acid toxicity. The likelihood of masking an incorrect diagnosis disappeared during the latter half of the last century, with the introduction of specific assays for folate and B12 deficiency, and with the ready availability and common use of B12 therapy.

A separate but related concern was that folic acid fortification might reduce the occurrence of macrocytic anaemia in vitamin B12 deficient individuals, and hence delay diagnosis of the deficiency. However, B12 deficiency has been shown to present without a macrocytic anaemia in 28% of cases in one study [18]. It follows that a macrocytic anaemia is not, and should not be regarded as, a requirement for the diagnosis of B12 deficiency. Again, with the advent of reliable assays for B12 deficiency and the clinical necessity of measuring a person’s B12 level if early neurological symptoms arise, concerns over the correct diagnosis were no longer an issue. This was acknowledged by the US IOM report [15] and was also the conclusion of an earlier assessment [19].

### Is the tolerable upper intake level (UL) of folate justified?

In the absence of treatment with B12 supplements, the progression of a B12 deficiency neuropathy is to be expected and is not evidence that folic acid is a cause of neurological damage. Furthermore, the IOM misinterpreted data from the 23 studies that they considered, with respect to the dose of folic acid thought to exacerbate the neurological progression. To our knowledge, this error has not previously been reported.

The IOM [15] considered the following two observations from the 23 studies:

1. At doses of folate of 5 mg/day and greater, there were more than 100 reported cases of neurological progression.
2. At doses of less than 5 mg/day of folate (0.33 to 2.5 mg/day), there were only eight reported cases.

Comparison of the “over 100 cases” with doses above 5 mg/day with only eight cases using doses below 5 mg/day was interpreted to mean that 5 mg/day should be the cut-off above which there was a risk of “harm” (i.e. exacerbation of the B12 neuropathy), and that 5 mg/day should be the LOAEL for folate. The argument rested on patients receiving “lower dose” treatments being taken to represent natural progression of the disease in the absence of B12 therapy while the proportion of progression among patients receiving higher doses was assumed to indicate folic acid “exacerbation”. However, the IOM analysis is incorrect.

**Fig. 4:**
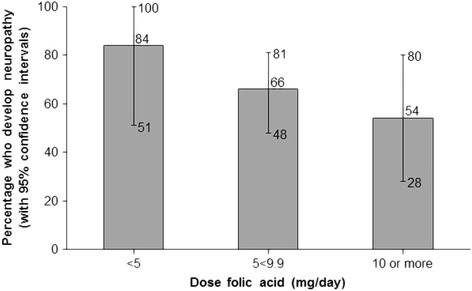
Neuropathy in patients with B12 deficient anaemia erroneously given folic acid according to dose [15]

Of the 23 studies considered by the IOM, 3 included patients taking a dose between 0.33 and 2.5 mg/day, 17 studies included patients taking a folic acid dose of 5 mg/day or greater, and 3 studies included some patients taking the higher dose, and some taking the lower. In the 23 studies 12 patients took daily folic acid in the lower dose category, and 8 developed neuropathy, i.e. 67% (95% CI 35%–90%). In contrast, 279 patients took folic acid in the higher dose category, and 147 developed neuropathy, i.e. 53% (95% CI 47%–59%). The rate of disease progression was no greater in patients taking higher doses of folic acid. Figure 4 uses the same data in an improved analysis with three dose categories instead of two - a meta-analysis of the proportions developing neuropathy in each study using the Freeman-Tukey transformation to allow for extreme estimates of variances in small studies and a random effects model to take account of the heterogeneity between the studies. This shows a non-significant decline in the proportion of patients developing neuropathy with increasing folic acid dose. There is no evidence or even a suggestion of a dose-response increase. The illogicality of attributing neurological toxicity to folic acid rather than the continued deficiency of B12, together with the absence of a folic acid dose-response effect, indicates that there is no evidence for an LOAEL for folate, and consequently no basis for a UL, which was arbitrarily taken as 20% of the LOAEL. No ULs have been set for vitamins B1, B2, B5, or B12, and there is no justification to set one for folic acid a water-soluble vitamin that is readily excreted.

## The perception of a conflict of policies

The IOM’s interpretation of old and limited medical and scientific evidence, before the ready availability of techniques for differential diagnosis, has hampered the introduction of mandatory fortification in some countries, in spite of the substantial evidence of efficacy and benefit. Progress in the consideration of fortification is being impeded by the acceptance of a tolerable upper intake level of folate at 1 mg a day. Any fortification programme designed to increase folic acid intake by, for example, 0.2 mg a day will necessarily increase folic acid intake throughout the population. This means that those people already consuming relatively high levels of folate might have their intake increased above the UL of 1 mg a day.

**Fig. 5:**
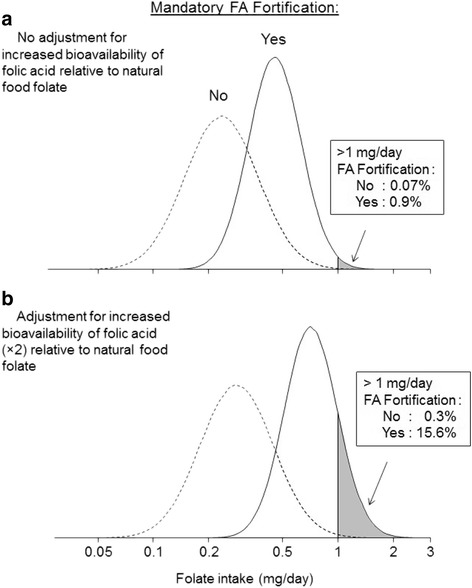
Distributions of folate intake with and without mandatory folic acid (FA) fortification (mean intake from mandatory fortification 0.2 mg/day) with (**b**) and without (**a**) adjustment for folic acid bioavailability. The percentage of people with a folate intake > 1 mg/day is shown in the boxes with and without mandatory fortification. Distributions include background intake from natural food folate and voluntary folic acid fortification. [Data on the usual intake of natural food folate and voluntary folic acid fortification in the UK from the National Diet and Nutrition Survey (NDNS 2008/9–2013/14) [20]. Almost identical distributions are obtained using the usual intake of folate in the USA from the Continuing Survey of Food Intakes by Individuals [21]. Intake from mandatory folic acid fortification assumed to be independent of natural food folate and folic acid from voluntary fortification but with the same population variance]

Figure 5 shows the effect of mandatory folic acid fortification (0.2 mg/day) on the distributions of total folate intake, both unadjusted and adjusted for the increased bioavailability of folic acid relative to food folate (Figs. 5a, b, respectively), based on data on the usual intake of dietary folate in the UK and USA [20, 21]. Figure 5 shows that, as a result of mandatory fortification, about one in six (15.6% estimated) might exceed a folate intake of 1 mg/day if the bioavailability of folic acid is considered.

**Fig. 6:**
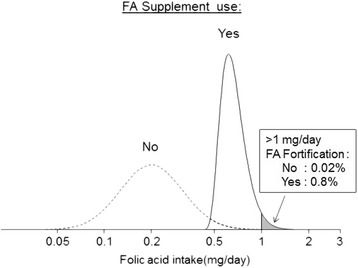
Distributions of folic acid (FA) intake with voluntary and mandatory fortification with and without 0.4 mg/day FA supplement (data on the usual intake of natural food folate and voluntary folic acid fortification in the UK from the National Diet and Nutrition Survey (NDNS 2008/9–2013/14). [20]. Almost identical distributions are obtained using the usual intake of folate in the USA from the Continuing Survey of Food Intakes by Individuals (CSFII) [21]. Intake from mandatory folic acid fortification assumed to be independent of natural food folate and folic acid from voluntary fortification but with the same population variance. Folic acid supplement assumed to be taken every day)

Figure 6 shows the distributions of folic acid intake (in contrast to folate intake shown in Fig. 5). The figure focuses on folic acid because the misplaced concern over possible toxicity (which led to an upper intake limit being set) relates to folic acid intake, not natural food folate intake, although, illogically, in setting the upper limit, the two were combined. The figure shows that taking a daily 0.4 mg folic acid supplement against a background of mandatory food fortification, still leads to some people (1% in our illustration) exceeding the 1 mg per day limit.

Figures 5 and 6 illustrate how mandatory fortification of flour with folic acid, so as to increase average intake to a level that protects the majority of women against NTDs, inevitably leads to a somewhat greater proportion of people exceeding the arbitrary 1 mg ceiling. It creates an unjustified conflict of policies, increasing folic acid intake to prevent NTDs but not doing so if it exceeds the 1 mg/day ceiling. As we have shown, the interpretation of the data used to set a UL for folic acid was flawed; a UL for folic acid is not needed and consequently the conflict of policies disappears.

## Withholding a benefit is a harm

A matter of public health concern is that some authorities, such as the European Commission Scientific Committee on Food, put greater weight on the hypothetical possibility of harm than on the proven evidence of benefit, apparently ignoring the fact that withholding a benefit is itself a harm. The EU report does not cite the MRC Vitamin Study randomised trial which demonstrated the benefit of increasing folic acid intake, but focuses almost entirely on hypothetical harms. The EU report states:

“Although there is no conclusive evidence in humans, the Committee concludes that the risk of progression of the neurological symptoms in vitamin B12 deficient patients as a result of folic acid supplementation cannot be excluded and should be considered the most serious adverse effect. In nearly all studies showing neurological relapse, dose levels < 5 mg folic acid per day have been applied and data on the effect of dose levels between 1 and 5 mg is limited to a few cases.” [10].

The opening sentence concedes that there is “no conclusive evidence” of neurological toxicity of folic acid [10]. Indeed, it would be more accurate to remove the word “conclusive”, for there is no evidence at all. Moreover, as we show above, the conclusion that “higher dose” folic acid is neurotoxic is based on a flawed analysis of uncontrolled observational studies and hence without evidential value. Furthermore, the report adopts an arbitrary five-fold reduction of an already unwarranted 5 mg/day toxicity level to 1 mg/day.

## Setting public health policy

As reasoned above, masking the diagnosis of B12 deficiency is an outdated concept (if ever it was a useful one), and the contention that folic acid is neurotoxic is scientifically wrong. Neither should be the basis for determining policy on folic acid fortification. To the extent that B12 deficiency is itself a public health concern [22] flour fortification should include B12 as well as folic acid, as has been done in the territory controlled by the Palestinian Authority.

The correct public health policy message is simple: flour should be fortified with folic acid so that, on average, folic acid intake is increased by at least 0.2 mg a day and preferably by about 0.4 mg, as was done in Chile, resulting in an approximate halving in the pregnancy prevalence of NTDs [7]. The use of an upper intake level for folate should be abandoned. We suggest that there are grounds for the US Institute of Medicine to reconsider its opinion on this issue in the light of the evidence and reasons given in this paper.

Failure to fortify is more than a missed opportunity; it is a tragedy. Since 1991, it has been estimated that there have been over five million preventable NTD pregnancies in the world [23]. The number of NTD pregnancies that could have been prevented by folic acid far exceeds the total number of cases of thalidomide induced phocomelia (10,000) [24]. While the thalidomide tragedy prompted immediate worldwide public health intervention, many countries still ignore the preventable toll of disability, stillbirth, infant death, and terminations of pregnancy caused by NTDs.

## Conclusion

There is no scientific basis for setting an upper level of intake for folate. Having such a limit, which has been set at 1 mg per day, has acted as a barrier to the wider introduction of mandatory fortification of flour with folic acid to prevent neural tube defects. For both reasons, the upper limit should be discarded. This would have the practical effect of leaving no scientific obstacle to the introduction of mandatory folic acid fortification in all countries, which would have an important global impact on the prevention of neural tube defects.
